# Aorto-left ventricular tunnel with anomalous origin of right coronary artery and bicuspid aortic valve: a case report

**DOI:** 10.1186/s13019-018-0770-1

**Published:** 2018-06-28

**Authors:** Xiaochun Ma, Jinzhang Li, Qian Zhang, Xiangqian Kong, Guidao Yuan, Zhengjun Wang, Chengwei Zou

**Affiliations:** 1 Department of Cardiovascular Surgery, Shandong Provincial Hospital affiliated to Shandong University, No.324 Jingwu Road, Shandong, 250021 People’s Republic of China; 2 School of Medicine, Shandong University, No.44 West Wenhua Road, Jinan, Shandong 250012 People’s Republic of China; 3 Department of Vascular Surgery, Shandong Provincial Hospital affiliated to Shandong University, No.324 Jingwu Road, Shandong, 250021 People’s Republic of China

**Keywords:** Aorto-left ventricular tunnel, Anomalous origin of right coronary artery, Bicuspid aortic valve

## Abstract

**Background:**

Aorto-left ventricular tunnel (ALVT) is a rare congenital extracardiac channel that connects the ascending aorta to the left ventricle. To our knowledge, no case has been thus far reported as ALVT with both anomalous origin of right coronary artery (AORCA) and bicuspid aortic valve (BAV).

**Case presentation:**

We reported a case of a 5-year-old female diagnosed as ALVT with accompanying AORCA and BAV which had been previously misdiagnosed as aortic regurgitation (AR) triggered by BAV. Additionally, a special modality of ALVT was confirmed in this case during the surgery in which the tunnel was formed by the separation between the roots of two aortic leaflets during the diastolic period.

**Conclusions:**

ALVT with both AORCA and BAV is clinically uncommon and the aberrant tunnel in ALVT can be formed by the gap between the roots of two aortic leaflets. Besides, ALVT with BAV might easily lead to an inaccurate diagnose as aortic regurgitation caused by BAV. Cardiac surgeons should be alerted for differential diagnosis of ALVT with BAV and isolated bicuspid aortic valve (BAV) causing aortic regurgitation (AR).

## Background

Aorto-left ventricular tunnel (ALVT) is an unusual congenital cardiac deformation in which an aberrant tunnel linking aortic root and left ventricle might trigger a varying presentation of left ventricle dilation and heart failure from in-utero fetal death to asymptomatic adulthood [1]. In this case report, we presented a special case of 5-year-old female patient with ALVT and concomitant anomalous origin of right coronary artery (AORCA) and bicuspid aortic valve (BAV).

## Case presentation

**Fig. 1:**
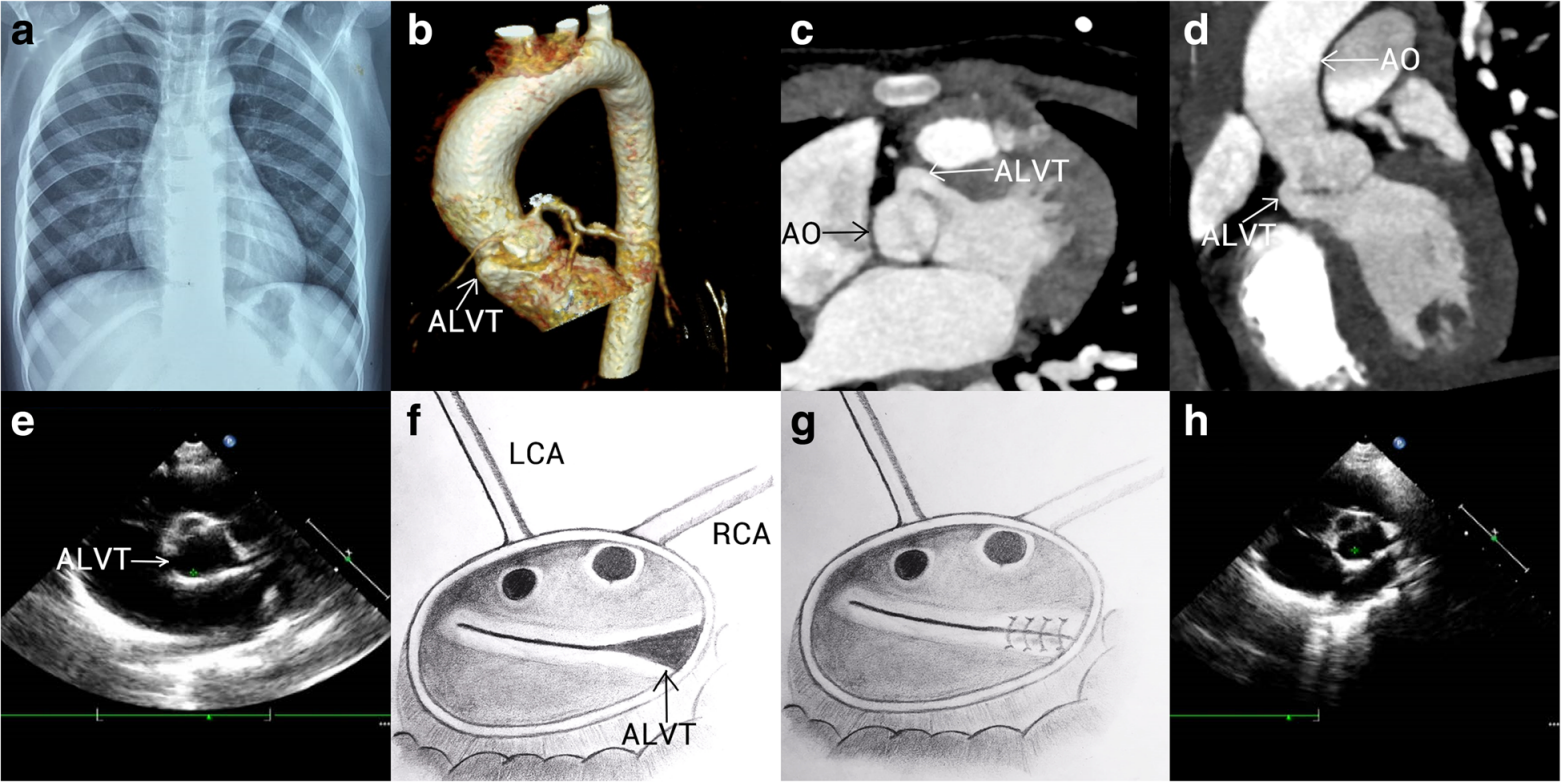
**a** The chest X-ray showed no significant aberrance. **b**-**e** The preoperative cardiac CTA, 3D reconstruction of cardiac CTA and echocardiography revealed an abnormal tunnel between the right posterior sinus and left ventricle. **f**-**g** Near anterolateral commissure the gap between the roots of two leaflets with a width of 0.6 cm formed into a special ostium with the inner wall of aorta when the leaflets were closed, which was sutured using 5–0 running polypropylene. **h** The postoperative echocardiography showed that the ALVT was turned off

A 5-year-old asymptomatic female was hospitalized in our center for surgical intervention for ALVT with AORCA and BAV. Tracing back to nearly 1 year, this entity of congenital cardiac abnormalities was accidentally detected by a routine echocardiography at a local hospital, with a misleading preliminary diagnosis as BAV with accompanying aortic regurgitation (AR). The patient and her parents reported no evident symptoms, concomitant congenital dysplasia, noteworthy past medical history and family history of inherited cardiac defects. Physical examination showed a grade I*V*/VI diastolic murmur at auscultatory area of aortic valve. Detailed results from a repeated echocardiography demonstrated: 1) an abnormal tunnel communicating the aortic root and left ventricle, with the opening of aortic segment at the level of the right posterior sinus (0.63 cm at width) and the opening of ventricular segment at the level of membranous interventricular septum (0.43 cm at width); 2) BAV without evident aortic stenosis or AR; 3) the left and right coronary arteries both originating from the left anterior sinus; 4) an enlarged left ventricle (LV) with normal left ventricular function (LV ejection fraction (LVEF) estimated as 65%). The chest X-ray found no significant aberrance (Fig. 1a). Additionally, a cardiac CTA further confirmed the diagnosis and revealed an abnormal tunnel between the right coronary sinus and left ventricle (Fig. 1b, c, d and e). Because the patient presented no obvious symptoms, no medical treatment was perfomed before the surgery.

The patients underwent the surgical repair for ALVT under general anesthesia and cardiopulmonary bypass (CPB). After a median sternotomy was performed and CPB was established, cardiac arrest was achieved using antegrade cardioplegia infusion at the root of aorta for myocardial protection. Subsequent to the horizontal incision at the aortic root, a good view for those anatomical abnormalities was obtained. Except that the AORCA and BAV were confirmed by the surgery, the aortic opening of the tunnel was clearly observed. Near anterolateral commissure the separation between the roots of two leaflets formed into a special ostium with the inner wall of aorta (0.6 cm at width) when the leaflets were closed (Fig. 1f). The gap between the roots of two leaflets was sutured using 5–0 running polypropylene to turn off the tunnel (Fig. 1g). Transesophageal echocardiography during the surgery showed no obvious AR. The surgical course went smoothly and the vital signs of the patient remained stable during the operation. No problem during cardioplegia and protection occurred. The CPB time and aortic clamping time were 103 and 75 min respectively.

Nearly 6 h after the surgery, tracheal intubation was weaned sucessfully. During the postoperative period, the continuous intravenous pumping of low-dose dopamine lasted for 7 h without other inotropic drugs administrated. The patient experienced an uneventful recovery without any complication and was discharged home one week after the operation. Echocardiography at discharge demonstrated the trivial AR and normal LV function with a LVEF of 65% (Fig. 1h). A long-term follow-up for the patient is highly recommended to detect the potential AR and AS caused by BAV in the future.

## Conclusions

ALVT is a rare congenital cardiac malformation with a reported incidence of less than 0.1–0.5% [1–3]. Clinical onset and progression of heart failure ranges from birth to adulthood, with a majority of the patients developing symptoms in their first year [4]. Associated anomalies of ALVT include proximal coronary abnormalities like coronary ostium originating within the tunnel or atresia of coronary ostium, and aortic valve abnormalities such as dysplastic or bicuspid valve with stenosis [5]. To our knowledge, no case has been so far reported as ALVT with both AORCA and BAV. Particularly in this case, the tunnel is formed by the gap between the aortic valve leaflets and the aortic wall. And we selected a direct suture instead of repair by patch, in order to avoid potentially impeding the opening of aortic valve. Besides, ALVT with BAV, which is clinically uncommon, might easily lead to an inaccurate diagnose as common AR caused by BAV. This misdiagnosis could result in the false clinical decision, delayed treatment and aggravated heart function. Thus cardiac surgeons should be alerted for differential diagnosis of ALVT with BAV and isolated BAV causing AR.

## Abbreviations

ALVT: Aorto-left ventricular tunnel
AORCA: Anomalous origin of right coronary artery
AR: Aortic regurgitation
AS: Aortic stenosis
BAV: Bicuspid aortic valve
CPB: Cardiopulmonary bypass
CTA: Computed tomography angiography
LV: Left ventricle
LVEF: LV ejection fraction
